# Supplementary material for: Risk of introduction and establishment of alien vertebrate species in transboundary neighboring areas
Source: Nat Commun. 2024 Jan 29;15:870. doi: 10.1038/s41467-024-45025-4 (PMC10824721; doi:10.1038/s41467-024-45025-4)
Supplement: Supplementary file 1 — Supplementary Information [file 41467_2024_45025_MOESM1_ESM.pdf]

Supplementary information

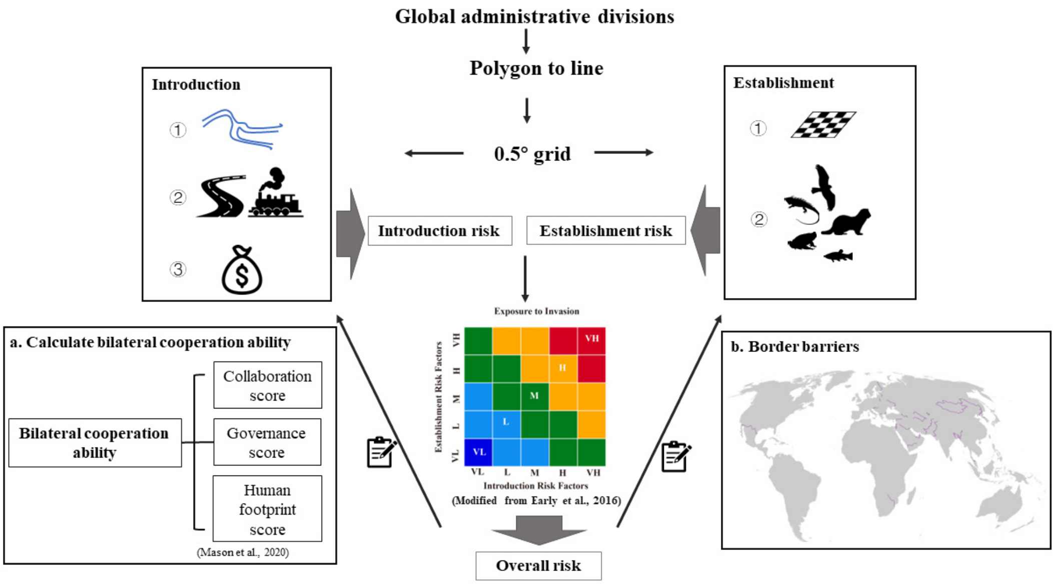

Supplementary Fig. 1 Analysis diagram to quantify the risk of introduction and establishment of alien vertebrate species across global borders.

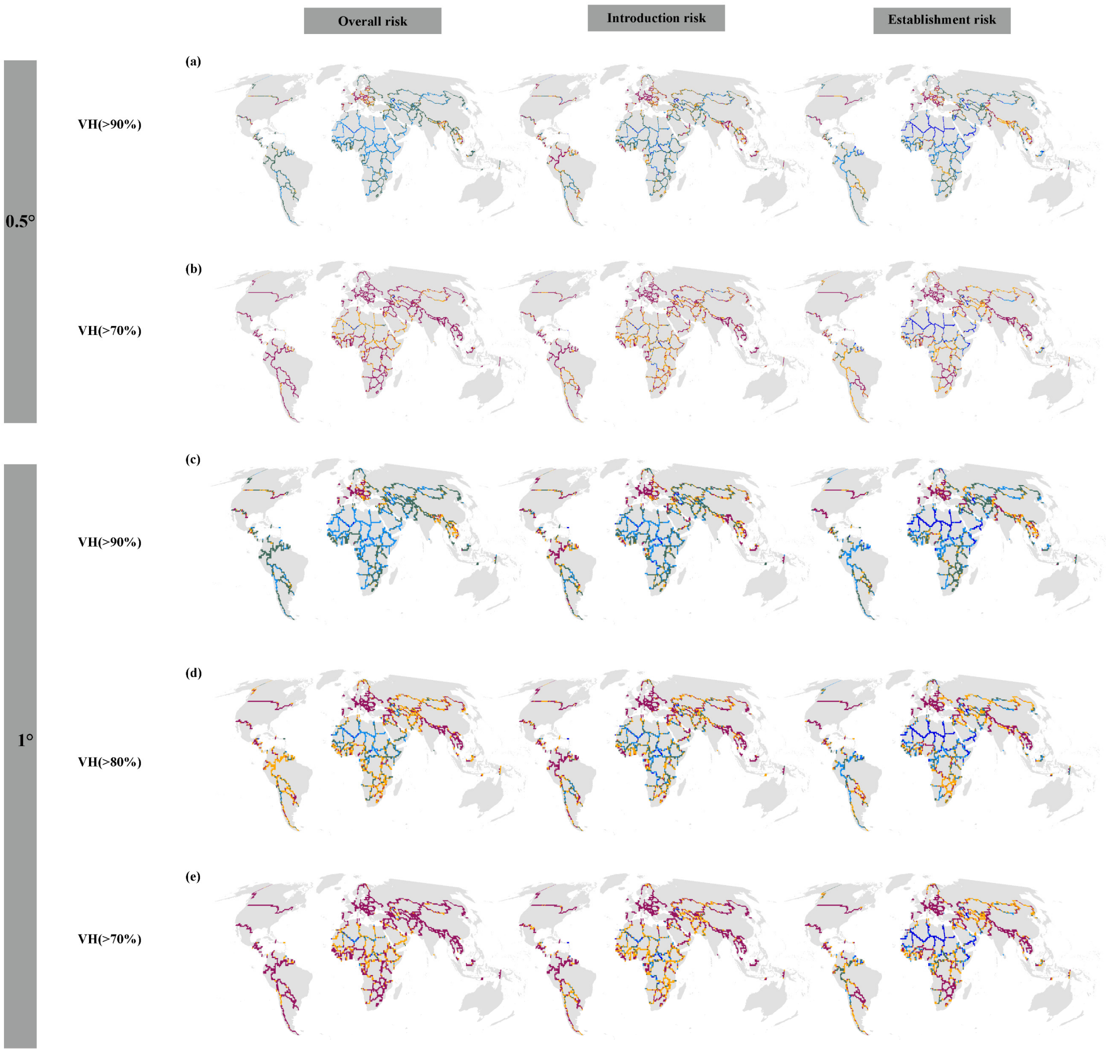

Supplementary Fig. 2 Introduction, establishment and overall invasion risks of alien vertebrate species across global borders were quantified at different grid-cell sizes and percentiles defining the highest invasion risk. (a), (b) showed the result with grid-cell sizes chosen at 0.5°, and the risk map using grid-cell sizes at 1° showing in (c), (d) and (e). Percentiles defining the highest risks (VH level) using 3 breakpoints (i.e., >90%, >80%, >70%).

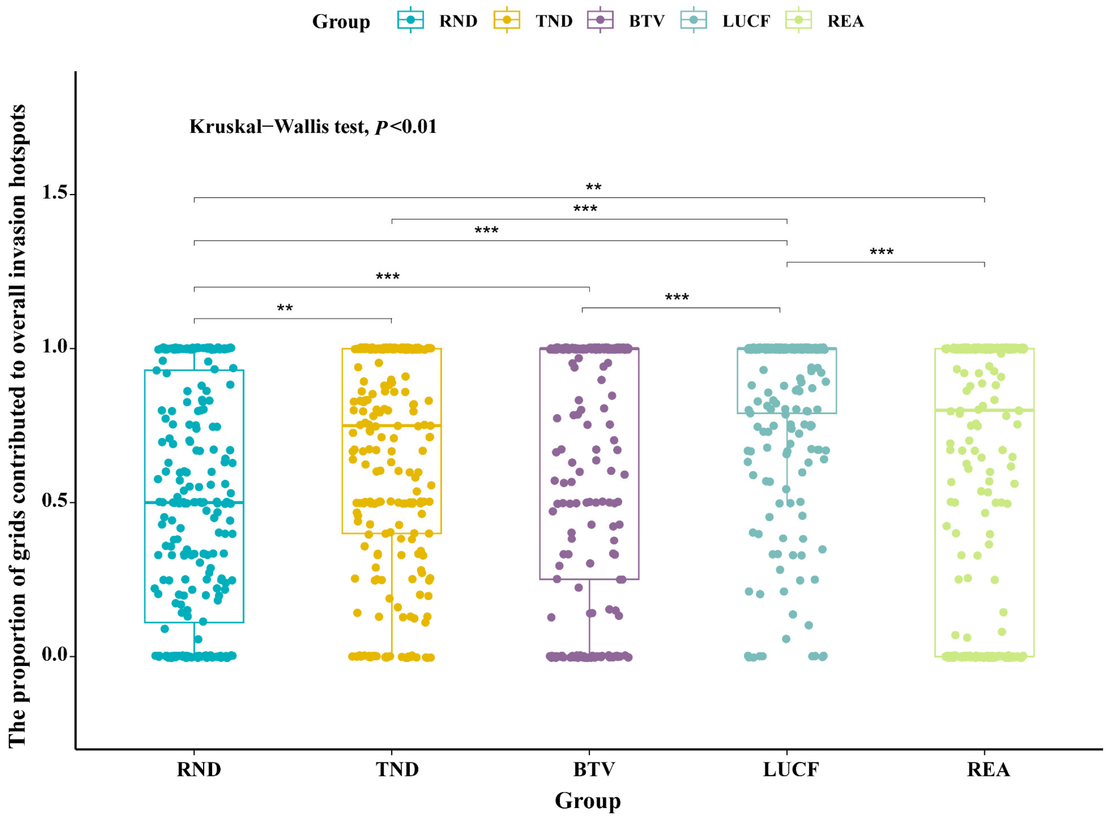

Supplementary Fig. 3 The importance of factors contributing to overall invasion hotspots of alien vertebrate species across global borders. The proportion of very high-risk (VH) grids of five variables contributing to the overall invasion hotspots (the grids with the highest level of overall risk) was compared using the Kruskal–Wallis test. The five variables include bilateral trade volume (BTV), traffic network density (TND), river network density (RND), land use change frequency (LUCF), and the richness of established alien vertebrates (REA).

(a)

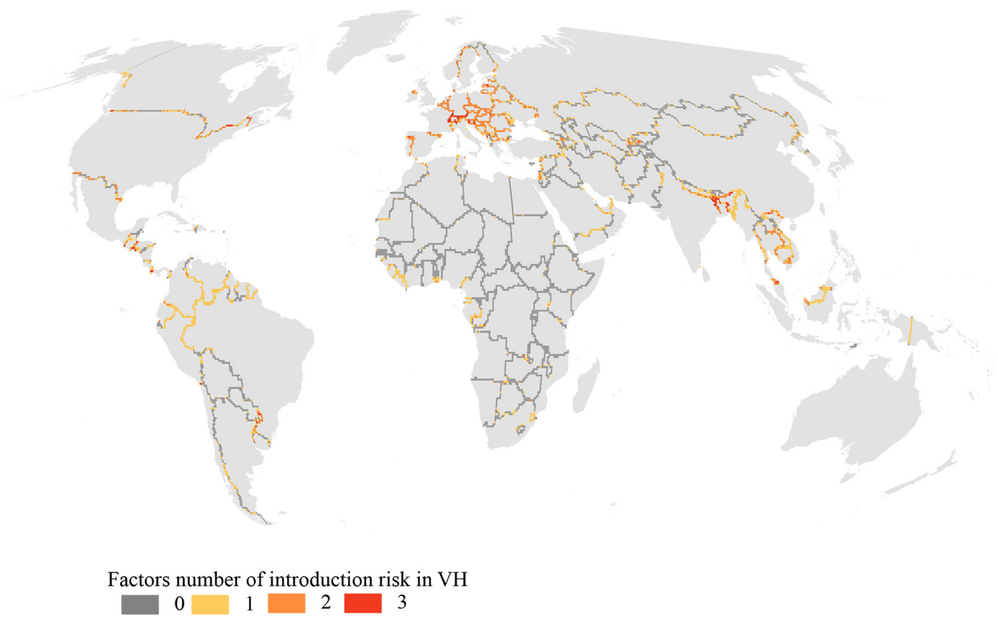

(b)

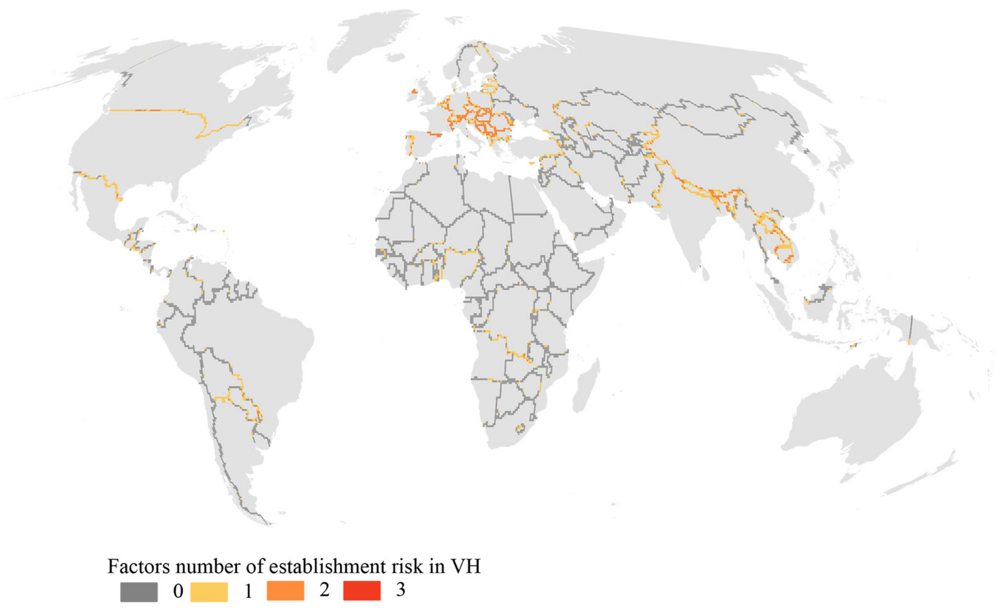

Supplementary Fig. 4 Spatial overlap of factors contributing to the very high levels (VH) of introduction risk (a) and establishment risk (b) of alien vertebrate species across global borders.

(a)

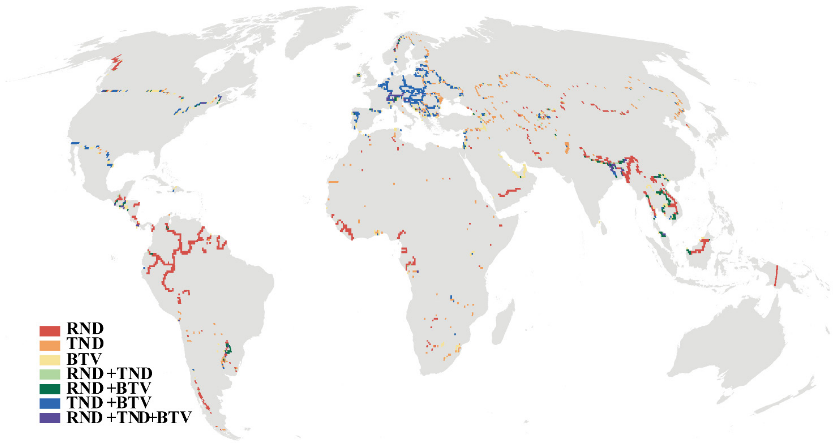

(b)

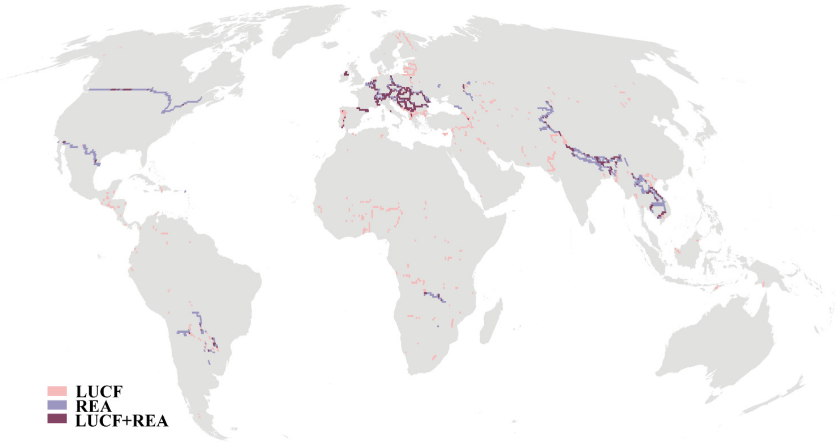

Supplementary Fig. 5 The exact factors driving the highest risk of introduction (a) and establishment (b) of alien vertebrate species across global borders. These factors include bilateral trade volume (BTV), traffic network density (TND), river network density (RND), land use change frequency (LUCF), and the richness of established alien vertebrates (REA).

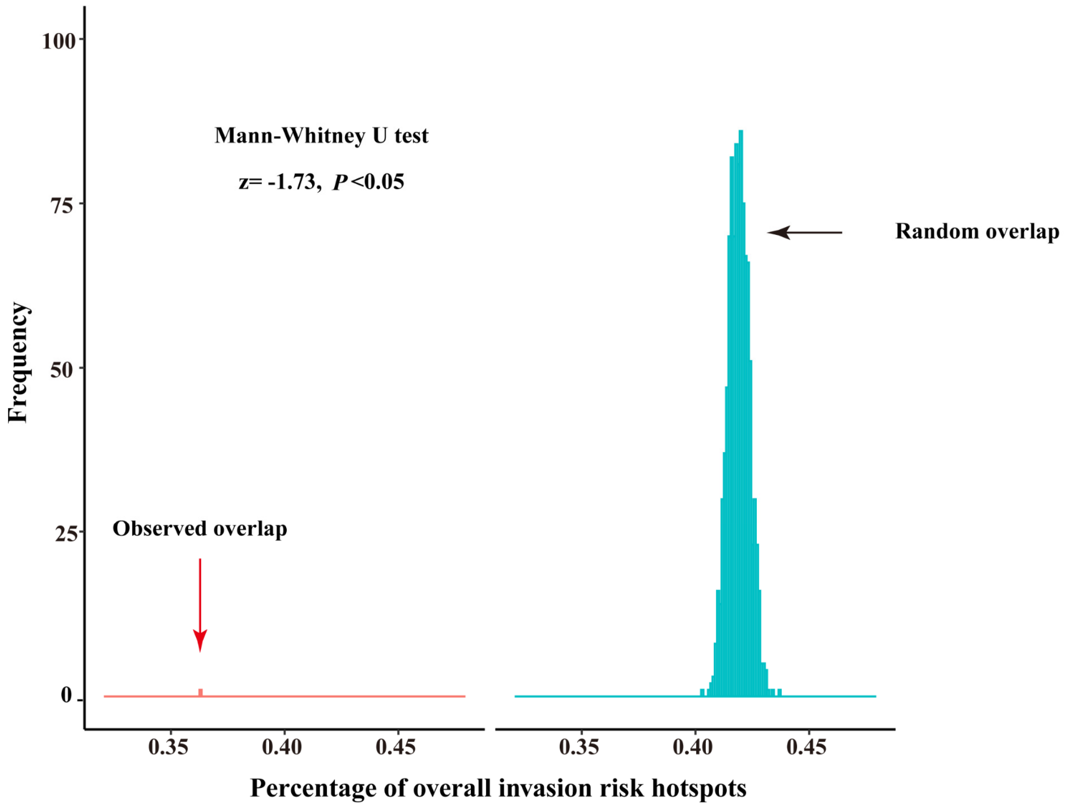

Supplementary Fig. 6 Comparison of spatial overlap between observed and null overlaps of high introduction and establishment grids of alien vertebrate species across global borders using the Mann–Whitney U test.

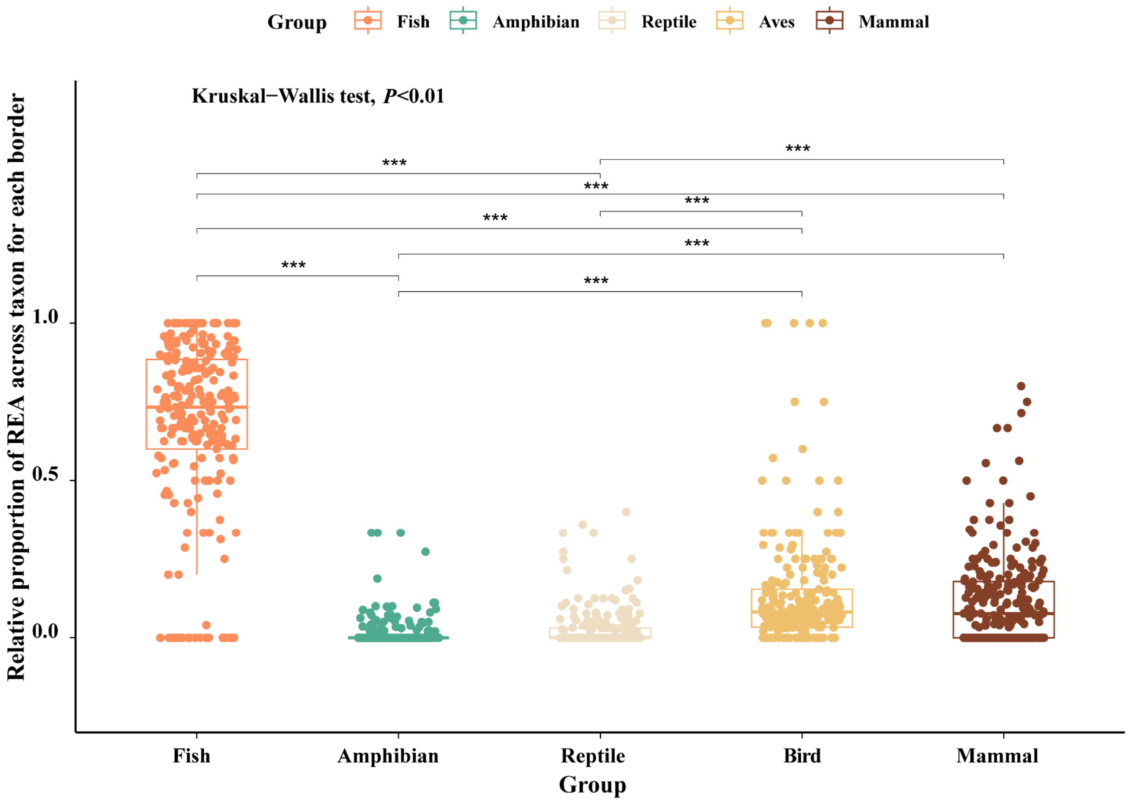

Supplementary Fig. 7 The relative importance of REA contributing to overall invasion hotspots (the grids with the highest level of overall risk) of alien vertebrate species across global borders determined by comparison of the relative proportion of REA across taxa along each border using the Kruskal–Wallis test.

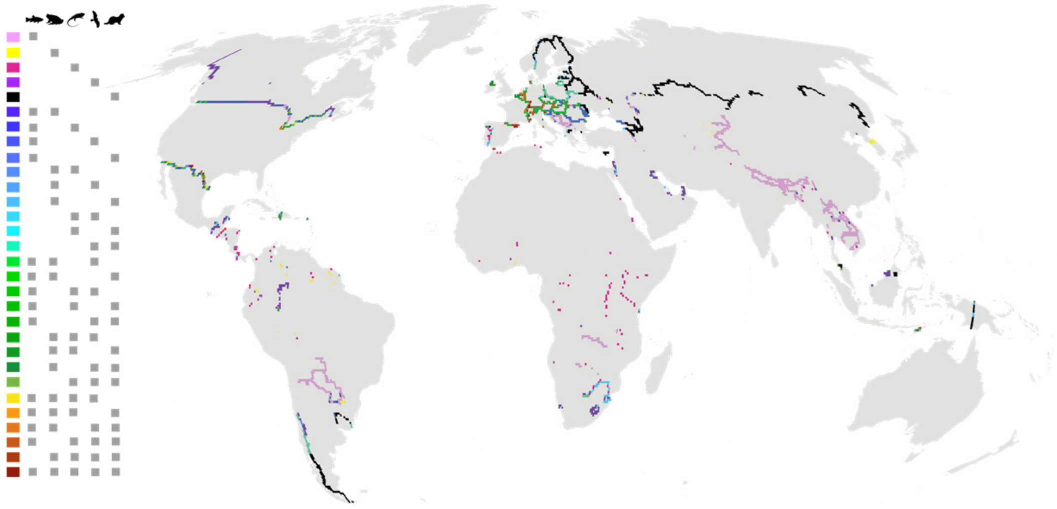

Supplementary Fig. 8 Spatial overlap of grids with the top 20% richness of established alien vertebrate species (REA) across global borders. Colors show different taxa combinations. Species silhouettes are sourced from PhyloPic (http://www.phylopic.org/).

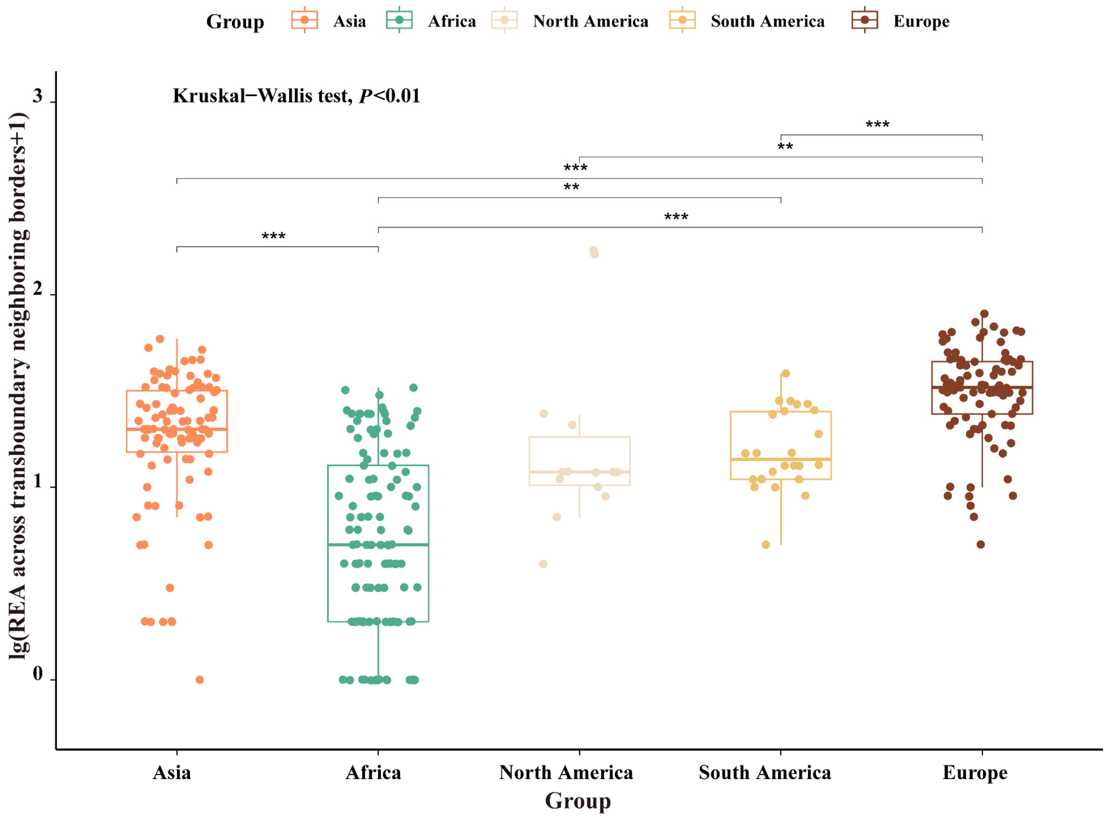

Supplementary Fig. 9 Spatial distribution of the richness of established alien vertebrate species (REA) across global borders among continents. The REA was compared across continents using the Kruskal–Wallis test.

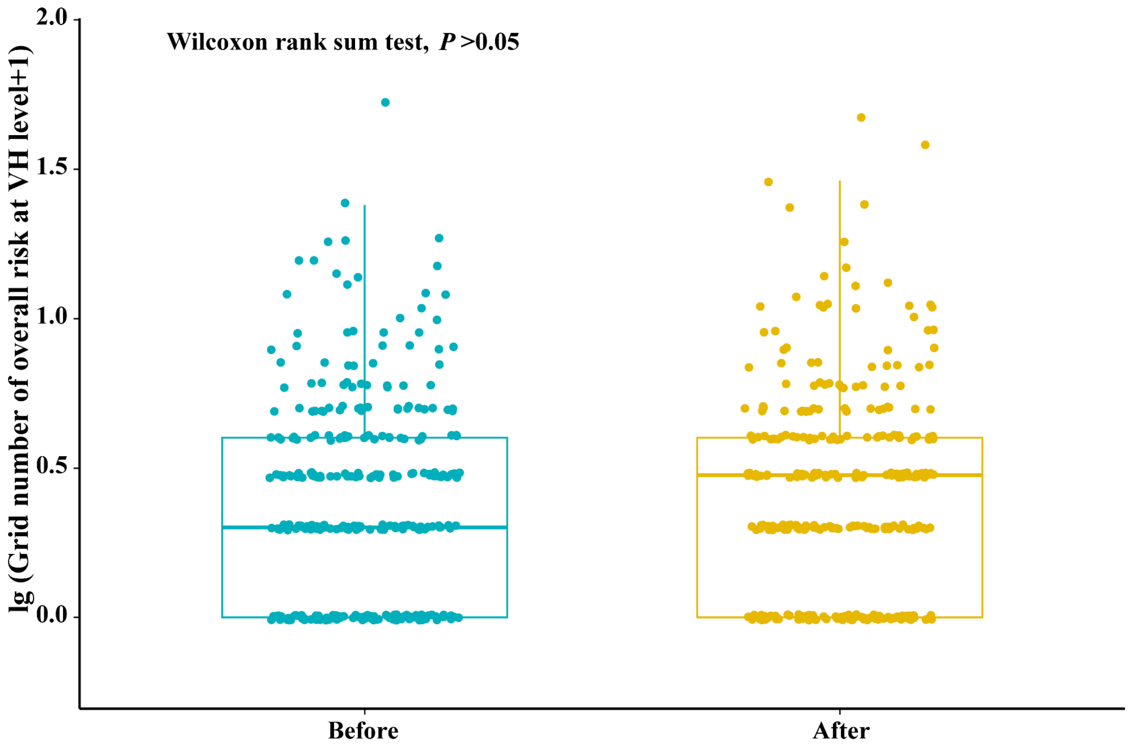

Supplementary Fig. 10 The significance test of the number of overall invasion hotspots (the grids with the highest level of overall risk) across global borders before and after accounting for sampling bias using the Wilcoxon rank sum test. The residuals of cross-taxon REA corrected by sampling effort were used to quantify the overall invasion risk of alien vertebrate species across global borders.

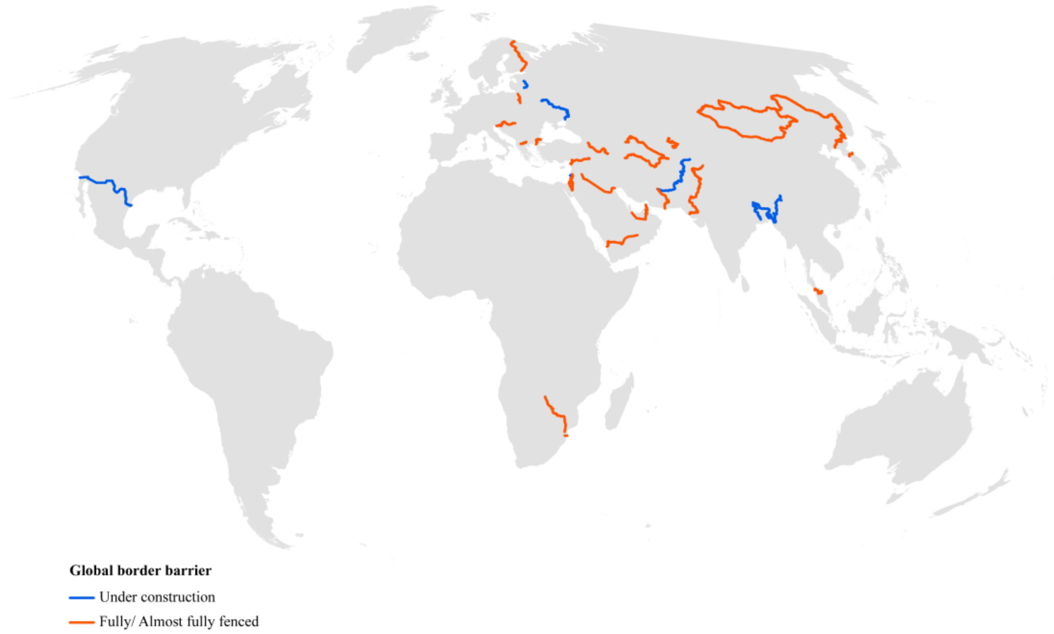

Supplementary Fig. 11 Locations of border physical barriers across global transboundary neighboring areas. Physical barrier data were based on Titley et al. (2021).

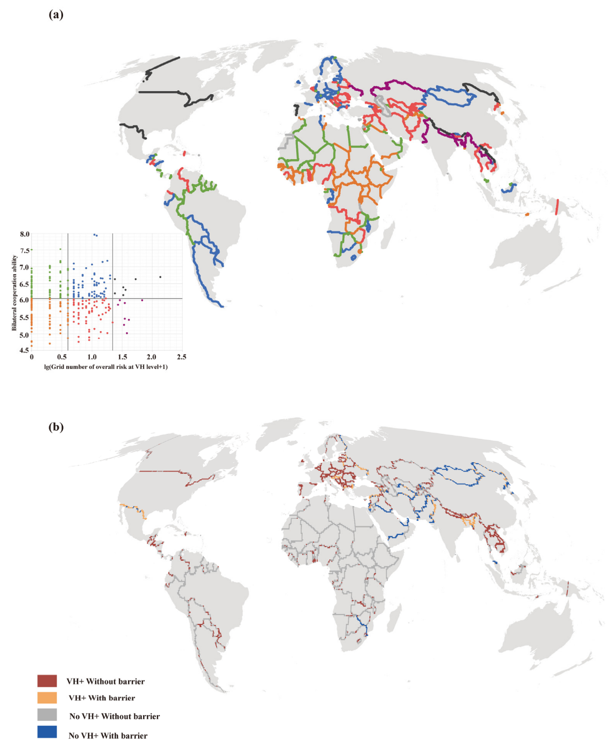

Supplementary Fig. 12 Spatial relationship between overall invasion hotspots (the grids with the highest level of overall risk) and potential uncertainties (i.e., bilateral cooperation ability and physical barrier). (a) Colors indicate borders with different numbers of overall invasion hotspots and different bilateral cooperation abilities. The lateral axis shows the median value of bilateral cooperation ability, and the two longitudinal axes show the median values and the first 5 % of grids with the overall highest-risk level. (b) The spatial distributions of borders with different combinations of the overall invasion hotspots and physical barrier presence: VH + Without barrier, VH + With barrier, No VH + With barrier, No VH + Without barrier. Global border barriers are presented in Supplementary Fig. 11. The number of overall invasion hotspots was log transformed before analysis to enhance normality.

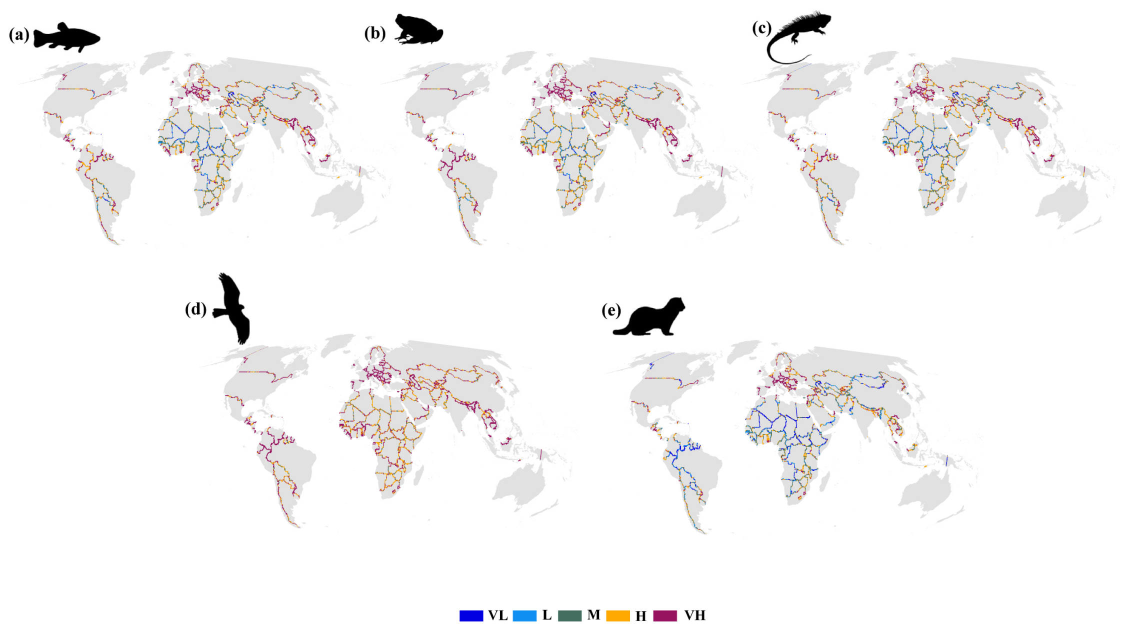

Supplementary Fig. 13 Introduction risk of alien vertebrate species across global borders using the exact important pathways for certain taxa. We incorporated river network density (RND) as a potential vector for (a) fish, (b) amphibians, (c) reptiles and (d) birds and used mountainous topographic heterogeneity (MTH) as a predictor for most taxa except birds.

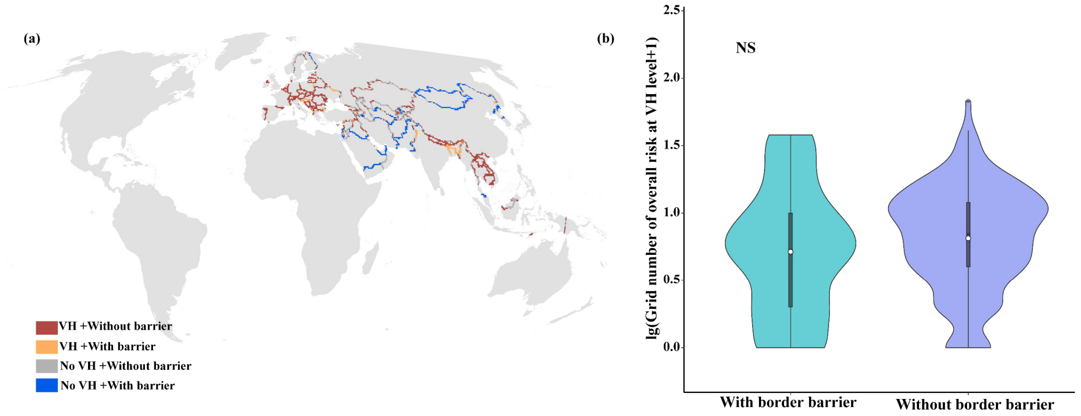

Supplementary Fig. 14 Overlap of overall invasion hotspots (the grids with the highest level of overall risk) and physical border barriers in Eurasia. (a) The spatial distributions of borders with different combinations of the overall highest invasion risk and physical barrier presence in Eurasia: VH + Without barrier, VH + With barrier, No VH + With barrier, No VH + Without barrier. (b) Comparison of the number of overall VH risk grids between borders with and without physical barriers showing that there is a low spatial overlap between grids with overall VH risk and the distribution of physical barriers in Eurasia (Mann–Whitney test, $P > 0.05$). Global border physical barriers are presented in Supplementary Fig. 11. The number of overall invasion hotspots was log transformed before analysis to enhance normality.

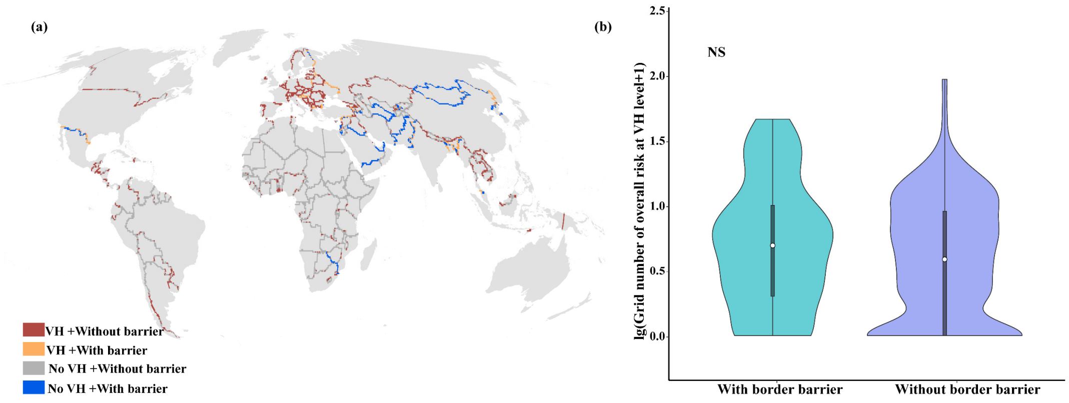

Supplementary Fig. 15 Overlap of overall invasion hotspots (the grids with the highest level of overall risk) and physical border barriers specific to reptiles and mammals. (a) The spatial distributions of borders with different combinations of the overall highest invasion risk and physical barrier presence in Eurasia: VH + Without barrier, VH + With barrier, No VH + With barrier, No VH + Without barrier. (b) Comparison of the number of overall VH risk grids between borders with and without physical barriers showing that there is a low spatial overlap between grids with overall VH risk and the distribution of physical barriers in Eurasia (Mann–Whitney test, $P > 0.05$). Global border physical barriers are presented in Supplementary Fig. 11. The number of overall invasion hotspots was log transformed before analysis to enhance normality.

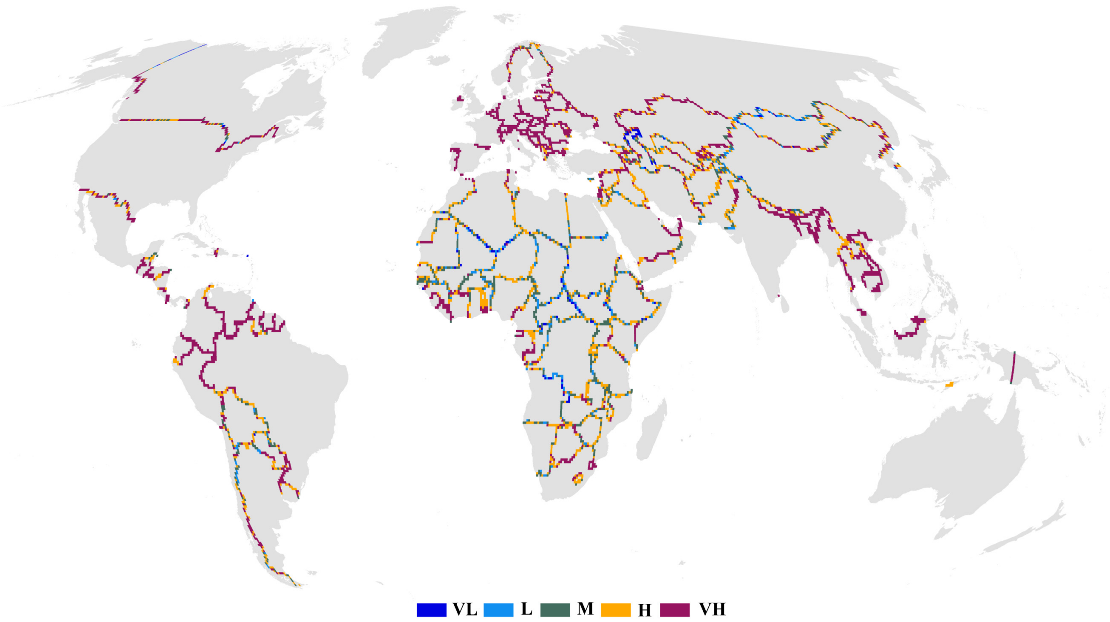

Supplementary Fig. 16 The introduction risk of alien vertebrate species across global borders after incorporating the airline route predictor.

Supplementary Table 1 Proportion of grids with different variables predicting the overall, introduction and establishment invasion hotspots of alien vertebrate species across global borders.

Variables predicting overall hotspots (1845)	Number of high-risk grids	Ratio
RND+LUCF	100	5%
RND+REA	65	4%
TND+LUCF	59	3%
TND+REA	43	2%
BTV+LUCF	45	2%
BTV+REA	47	3%
RND+TND+LUCF	53	3%
RND+TND+REA	35	2%
RND+BTV+LUCF	83	4%
RND+BTV+REA	55	3%
RND+LUCF+REA	58	3%
TND+BTV+LUCF	82	4%
TND+BTV+REA	171	9%
TND+LUCF+REA	43	2%
BTV+LUCF+REA	70	4%
RND+TND+BTV+LUCF	84	5%
RND+TND+BTV+REA	85	5%
RND+TND+LUCF+REA	31	2%
RND+BTV+LUCF+REA	97	5%
TND+BTV+LUCF+REA	297	16%
RND+TND+BTV+LUCF+REA	242	13%
**Variables predicting introduction hotspots (2189)**	**Number of high-risk grids**	**Ratio**
RND	737	34%
TND	408	19%
BTV	303	14%
RND+TND	50	2%
RND+BTV	131	6%
TND+BTV	460	21%
RND+TND+BTV	100	5%
**Variables predicting establishment hotspots (1623)**	**Number of high-risk grids**	**Ratio**
LUCF	616	38%
REA	616	38%
LUCF+REA	391	24%
