# Supplementary material for: Risk of introduction and establishment of alien vertebrate species in transboundary neighboring areas
Source: Nat Commun. 2024 Jan 29;15:870. doi: 10.1038/s41467-024-45025-4 (PMC10824721; doi:10.1038/s41467-024-45025-4)
Supplement: Supplementary file 3 — Description of Additional Supplementary Files [file 41467_2024_45025_MOESM3_ESM.pdf]

**Description of Additional Supplementary Files**

File Name: Supplementary Data 1
Description: The number of grids with overall, introduction, and establishment hotspots of alien vertebrate species across global borders contributed by different predictor variables.

File Name: Supplementary Data 2
Description: Number of grids with the top 20% richness of established alien vertebrate species in global borders across taxa.

File Name: Supplementary Data 3
Description: Database and literature used to collect the occurrence data of established alien vertebrate species across global borders. + indicates the database has been used to collect the distribution information for the species.

File Name: Supplementary Data 4
Description: Relative proportion of the richness of established alien vertebrate species across taxa in overall invasion hotspot grids of global borders.

File Name: Supplementary Data 5
Description: List of keywords for multilingual literature search to collect occurrence data of established alien amphibians, reptiles and fish species.
