# Supplementary material for: Risk of introduction and establishment of alien vertebrate species in transboundary neighboring areas
Source: Nat Commun. 2024 Jan 29;15:870. doi: 10.1038/s41467-024-45025-4 (PMC10824721; doi:10.1038/s41467-024-45025-4)
Supplement: Supplementary file 9 — Reporting Summary [file 41467_2024_45025_MOESM9_ESM.pdf]

nature portfolio

Corresponding author(s): Xuan Liu

Last updated by author(s): Dec 5, 2023

Reporting Summary

Nature Portfolio wishes to improve the reproducibility of the work that we publish. This form provides structure for consistency and transparency in reporting. For further information on Nature Portfolio policies, see our Editorial Policies and the Editorial Policy Checklist.

Statistics

For all statistical analyses, confirm that the following items are present in the figure legend, table legend, main text, or Methods section.

n/a	Confirmed	
☐	☒	The exact sample size (*n*) for each experimental group/condition, given as a discrete number and unit of measurement
☐	☒	A statement on whether measurements were taken from distinct samples or whether the same sample was measured repeatedly
☐	☒	The statistical test(s) used AND whether they are one- or two-sided<br>*Only common tests should be described solely by name; describe more complex techniques in the Methods section.*
☐	☒	A description of all covariates tested
☐	☒	A description of any assumptions or corrections, such as tests of normality and adjustment for multiple comparisons
☐	☒	A full description of the statistical parameters including central tendency (e.g. means) or other basic estimates (e.g. regression coefficient) AND variation (e.g. standard deviation) or associated estimates of uncertainty (e.g. confidence intervals)
☐	☒	For null hypothesis testing, the test statistic (e.g. *F*, *t*, *r*) with confidence intervals, effect sizes, degrees of freedom and *P* value noted<br>*Give P values as exact values whenever suitable.*
☒	☐	For Bayesian analysis, information on the choice of priors and Markov chain Monte Carlo settings
☒	☐	For hierarchical and complex designs, identification of the appropriate level for tests and full reporting of outcomes
☐	☒	Estimates of effect sizes (e.g. Cohen's *d*, Pearson's *r*), indicating how they were calculated

Our web collection on statistics for biologists contains articles on many of the points above.

Software and code

Policy information about availability of computer code

Data collection	The map data generated in this study using ESRI ArcGIS Pro v.2.5.2. and are available in the Figshare database. The processed box plot figure in this study are generated using R v.4.1.1.
Data analysis	Global transboundary maps of invasion risks were conducted using ESRI ArcGIS Pro v.2.5.2. Data analysis and plotting were processed with the“rstatix”, “ggpubr”, “ggplot2”, “tidyverse”, “ggthemes”, “viridis”, “hrbrthemes”, “readr”, “ggsignif”, “coin”, “PMCMR”, “dplyr”, “rsq”, “raster”, “spatialEco”, and “sf”, “PMCMRplus” packagesin R v.4.1.1.

For manuscripts utilizing custom algorithms or software that are central to the research but not yet described in published literature, software must be made available to editors and reviewers. We strongly encourage code deposition in a community repository (e.g. GitHub). See the Nature Portfolio guidelines for submitting code & software for further information.

Data

Policy information about availability of data

All manuscripts must include a data availability statement. This statement should provide the following information, where applicable:

- Accession codes, unique identifiers, or web links for publicly available datasets
- A description of any restrictions on data availability
- For clinical datasets or third party data, please ensure that the statement adheres to our policy

The processed figure data, database and literature used to collect the occurrence data of established alien vertebrate species, and keywords for multilingual

literature search to collect occurrence data of alien amphibians, reptiles and fish species are available at Supplementary Data. The map data generated in this study using ESRI ArcGIS Pro v.2.5.2. and are available in the Figshare database (https://doi.org/10.6084/m9.figshare.24751929.v1).

Research involving human participants, their data, or biological material

Policy information about studies with human participants or human data. See also policy information about sex, gender (identity/presentation), and sexual orientation and race, ethnicity and racism.

Reporting on sex and gender	N/A
Reporting on race, ethnicity, or other socially relevant groupings	To quantify bilateral cooperation abilities, we used a parameter known as "Bilateral cooperation ability", which took into account information on cooperation, governance, and human pressure based on Mason et al. (2020). The bilateral cooperation capacities were averaged for each country pairing.<br>We did not use race or ethnicity as a proxy for social variables.
Population characteristics	N/A
Recruitment	N/A
Ethics oversight	N/A

Note that full information on the approval of the study protocol must also be provided in the manuscript.

Field-specific reporting

Please select the one below that is the best fit for your research. If you are not sure, read the appropriate sections before making your selection.

☐ Life sciences ☐ Behavioural & social sciences ☒ Ecological, evolutionary & environmental sciences

For a reference copy of the document with all sections, see nature.com/documents/nr-reporting-summary-flat.pdf

Ecological, evolutionary & environmental sciences study design

All studies must disclose on these points even when the disclosure is negative.

Study description	Short geographic distance, similar social cultures, frequent human and trade exchanges make cross-border neighboring areas particularly sensitive to biological invasions worldwide. However, the invasion risk in global transboundary neighboring areas remains unknown. We provided the first global study to identify the invasion hotspots at transboundary neighboring areas by evaluating alien species introduction and establishment risks. We also disentangled the relative importance of different environmental and anthropogenic variables, such as RND(river network density), TND (traffic network density), BTV (bilateral trade volume), LUCF (land use change frequency), and REA (richness of established alien vertebrates) in predicting the potential invasion risks among 334 bilateral borders, which intersected with a total of 5,088 0.5° grids. In addition, we explored the spatial relationship between invasion risks and the bilateral cooperation abilities, and identified regions where the physical border barriers located that may complicate the transboundary invasion risks.
Research sample	We evaluated the invasion risk in transboundary areas by analyzing 334 pairs of bilateral regions, which resulted in a total of 5,088 0.5° grids.
Sampling strategy	We evaluated the invasion risk at transboundary areas based on a quantitative framework, which combined the relative likelihood of introduction and establishment of invasive alien species. To identified the hotspots of cross-border invasion risk, we collected information carefully from various widely used databases and published references. Global map of transboundary invasion risk were at a spatial resolution of 0.5° grids, which was widely accepted as appropriate for global scale studies as it balanced analysis precision and computational efficiency.
Data collection	Data of administrative designations was used from the Global Database of Administrative Areas v.3.4 (GADM). The bilateral trade volumes were obtained from the United Nations Commodity Trade Statistics Database (https://comtrade.un.org/). We collected the human population data from the United Nations database (https://population.un.org/wpp/Download/Standard/Population) and the NASA database (GPW, v4). The data for roads and railways were acquired from the Natural Earth database. The river distribution data obtained from the Mapping the world's free-flowing rivers database. The distribution and occurrence data of established alien fishes at the drainage basin level were collected based on Tedesco et al. (2017) and Su et al. (2021). Data on amphibians and reptiles were compiled from multiple publications, including Kraus (2015)'s compendium and Capinha et al. (2017)'s updates. Data on established alien birds were collected from the Global Avian Invasions Atlas. Data on established alien mammals were obtained from the global Distribution of Alien Mammals database (DAMA). Land use change frequency (LUCF) data were downloaded from 1960 to 2019 provided by Winkler et al. (2021). We quantified bilateral cooperation abilities modified from Mason et al. (2020). Physical barrier data were based on Titley et al. (2021). Data above recored by Qing Zhang, Weishan Tu, Shengnan Chen, Yanhua Hong, Lixia Han, Yuanbao Du, Shimin Gu and Zhiqiang Lin. The occurrence data of different established alien amphibians, reptiles and fish also collected from intensive literature reviews using different languages (English, French, Danish, Estonian, Finnish, German, Norwegian, Portuguese, Russian, Spanish, Swedish, and Mandarin Chinese), recorded by Qing Zhang, Yanxia Li, Jiajie Yu and Sadia Ashraf. Keywords data in literature search using different languages checked by Akbayan Yerlankyzy, Santiago Montero-Mendieta and Wenjie Li.

Timing and spatial scale	We collected the administrative designations information from the Global Administrative Areas (GADM) database in 2020. We obtained the bilateral trade volumes from the United Nations Commodity Trade Statistics Database from 2011 to 2020 on September 2021.We obtained the human population density data from NASA database (2020) at a resolution of 2.5 arcmin. The data for roads and railways were acquired from the Natural Earth database on September 2021. Data on established alien vertebrates were collected and conducted from database and reference on July 2022. Land use change frequency (LUCF) data were downloaded from 1960 to 2019 provided by Winkler et al. (2021).We quantified bilateral cooperation abilities modified from Mason et al. (2020). Physical barrier data were based on Titley et al. (2021).
Data exclusions	We excluded administrative regions that do not have contiguous border relations, such as Australia.
Reproducibility	All data sources have been provided in the supporting materials, and we will additionally make all original data public in a Dryad Digital Repository upon the publication of the work.
Randomization	We applied a null model approach by generating 1000 random distributions of high risk of introduction and establishment grids to test whether the spatial overlap between observed introduction and establishment risks was more concentrated in certain hotspot regions than that would be expected by chance based on the Mann–Whitney U test.
Blinding	Blinding was not relevant with our study because all data used in the present study was collected from public databases and published references, which have been provided in the supporting material.

Did the study involve field work? ☐ Yes ☒ No

Reporting for specific materials, systems and methods

We require information from authors about some types of materials, experimental systems and methods used in many studies. Here, indicate whether each material, system or method listed is relevant to your study. If you are not sure if a list item applies to your research, read the appropriate section before selecting a response.

Materials & experimental systems

n/a	Involved in the study	
☒	☐	Antibodies
☒	☐	Eukaryotic cell lines
☒	☐	Palaeontology and archaeology
☒	☐	Animals and other organisms
☒	☐	Clinical data
☒	☐	Dual use research of concern
☒	☐	Plants

Methods

n/a	Involved in the study	
☒	☐	ChIP-seq
☒	☐	Flow cytometry
☒	☐	MRI-based neuroimaging
